# Proper name anomia in people with Alzheimer’s disease: implications for diagnosis and treatment—a systematic review

**DOI:** 10.1038/s44400-026-00058-y

**Published:** 2026-02-02

**Authors:** Aygun Badalova, Joshua Stott, Alex P. Leff

**Affiliations:** 1 UCL Queen Square Institute of Neurology, University College London, London, UK; 2 Institute of Cognitive Neuroscience, University College London, London, UK; 3 ADAPTlab, Research Department of Clinical, Educational and Health Psychology, London, UK

**Keywords:** Diseases, Neurological disorders, Neurodegenerative diseases, Alzheimer's disease, Parkinson's disease, Attention, Language, Dementia, Neurodegeneration, Neurodegenerative diseases, Neurovascular disorders, Parkinson's disease, Preclinical research, Cognitive ageing, Cognitive neuroscience, Learning and memory, Neural ageing

## Abstract

Proper name anomia (PNA) is a common experience that can become unpleasantly amplified in people with Alzheimer’s disease (AD). In this systematic review, we discuss the key cognitive stages where naming can fail: facial recognition, person-specific semantics, and proper-name retrieval. We examine claims that PNA is an early indicator of AD and review studies that have attempted to treat PNA in individuals with AD. Twenty-two eligible studies were included. The main findings are that individuals with AD frequently experience difficulties in recalling proper names at any point in the disease process, with the distribution of functional breakdown between the three key cognitive stages involved in successful naming being: facial recognition (19%), person-specific semantics (30%), and proper-name retrieval (40%). PNA can be an early manifestation of AD. Effective behavioural treatments are available for those whose naming difficulties occur at the retrieval stage, including trial-by-trial practice using vanishing cues and spaced retrieval. We also provide clinical recommendations regarding the diagnosis and treatment of PNA.

## Introduction

Dementia of the Alzheimer type or Alzheimer’s Disease (AD) contributes to 50-60% of all cases of the 55 million people worldwide living with dementia, a number predicted to rise to 139 million by 2050^1^. Although memory impairment is a hallmark of AD, language impairments, such as word-finding difficulties or anomia, are present in ~50% of people with mild/moderate AD and over 80% with a severe form^2^.

The areas of the brain most affected in the early stages of AD are the medial temporal lobe structures, notably the entorhinal cortex and hippocampus^3^. In addition, recent studies have highlighted that functional changes and early amyloid-β deposition precede or accompany structural decline in the posterior cingulate cortex, precuneus, and medial prefrontal cortex, key nodes of the brain’s default mode network. These regions exhibit altered connectivity patterns and metabolic dysfunction detected by PET and resting-state fMRI^4,5^, suggesting a broader functional network disruption in the prodromal phases of AD. Importantly, these brain regions support key cognitive processes such as episodic memory, self-referential thinking, and autobiographical recall^6,7^. Therefore, disruption within this network may contribute to the memory deficits that characterize the earliest symptomatic stages of Alzheimer’s disease^8^.

According to standard views of memory processing, the hippocampal complex plays a critical role in the establishment of new episodic and semantic memories by linking together cortical representations^9,10^. Over time, by rehearsal or reinstatement, connections are established within the cortex which become independent of the hippocampal consolidation.

There is, however, accruing evidence that slower, non-hippocampal dependent processes can support the learning of new semantic facts and vocabulary^11,12^. Anomia is primarily considered as an impairment in the retrieval of word-based neural representations^13,14^; however, another cause is damage to representations that the words relate to. When this happens at the semantic level (e.g. in semantic dementia), the abstract concepts themselves become eroded, and cuing methods to aid retrieval are generally unsuccessful^15^. In non-language-led forms of dementia, anomia is typically caused by difficulty in retrieving words, a symptom that is potentially amenable to practice-based cueing therapies^16^.

In linguistic philosophy, proper names are typically called rigid designators^17^. This means they consistently refer to the same object in all possible scenarios where that object exists and never refer to anything else (two different people can have the same name, but once we have established who we are talking about, that name refers to them and only them). Aligned with this rigidity is the randomness of proper names; although you are assigned your name, you can change it, which weakens the semantic associations of any proper name^18^.

These properties may explain why proper names are particularly hard to retrieve compared with other sources of personalised information, or other word classes^19^. It is commonly acknowledged that remembering proper names becomes notably more difficult with increasing age^19,20^.

Research has shown that proper nouns are more difficult to recall than common nouns because they are less semantically related to the objects they refer to, e.g. all apples have common features, whereas all people named ‘John’ do not. Studies have shown that even healthy older adults have more difficulty recalling proper nouns than common nouns.

Semenza et al. ^21^ in 2003 have suggested that proper nouns may be a more sensitive indicator of AD and that PNA may be a good way to distinguish very mild cases of AD and healthy older adults, where standardised clinical tests such as the Mini-Mental State Examination (MMSE) and the extended Milan Overall Dementia Assessment (MODA) fail to do this.

People with AD experience more prominent difficulty in recalling proper names compared to age-matched controls^22^ at all stages of dementia, which patients find annoying and embarrassing^23^. Patients with PNA are likely to feel frustrated and distressed, contributing to the loss of confidence in social situations, which might, in turn, result in withdrawal and isolation^24^. Forgetting people’s names can be an embarrassing issue, and this problem tends to worsen with normal aging and certain types of dementia. As individuals age or experience cognitive decline associated with dementia, the ability to recall names can be particularly affected, leading to increased difficulties in remembering and retrieving familiar names.

Due to a lack of standardised assessment tools, PNA is often not identified or treated in people with AD. Nonetheless, the treatment of PNA is of potential benefit both to the person with dementia and to their families and carers. It may help to improve communication, thereby increasing quality of life. This, in turn, may reduce stress for carers. There is also evidence suggesting that treatment may slow the progression of anomia in people with dementia over time, even affording some protective benefit to lexical items that are not yet lost^25^. Recent research also supports maintenance-based approaches, with Flurie et al.^26^ demonstrating that such interventions can prevent lexical dropout.

**Fig. 1:**
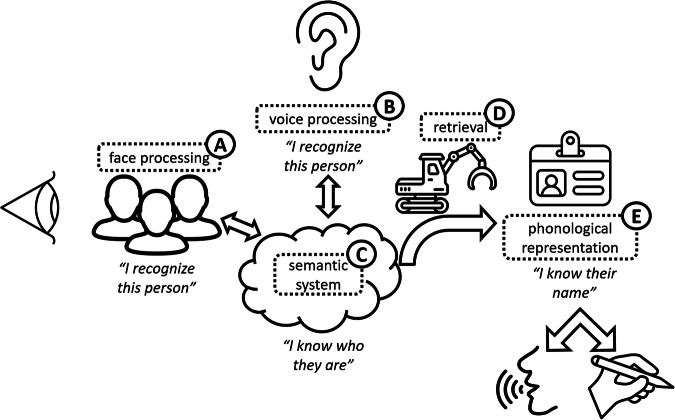
Diagrammatic representation of the cognitive processes involved in naming a familiar person. We generally recognise people from their face or voice. These recognition processes can break down independently, leading to (**A**) prosopagnosia and (**B**) phonagnosia^72^. Person-specific semantics can become eroded (**C**), most commonly in people with Semantic Dementia. Phonological representations can also be lost in certain forms of Primary Progressive Aphasia (**E**), but these conditions, and those with post-stroke aphasia, more often experience a failure of retrieval (**D**), and are therefore cueable.

The ability to name an image of a face can fail at three main cognitive stages: facial recognition, person-specific semantics and proper name retrieval (Fig. 1: influenced by similar models by Bruce and Young^27^, Valentine et al.^28^ and Gorno Tempini et al.^29^). In most models of successful naming, all three processes must operate, probably in a sequential fashion^27,30,31^. It has been suggested that naming can occur without access to person-specific knowledge^32^, but this has largely been refuted by the data, e.g., ref. ^33^. Most researchers and clinicians test PNA by using a series of images of ‘well-known’ people. The list of people who are well-known varies over cultures and time, leading researchers to have to continually update the Famous Faces Test, adapting it for individual countries or cultures^34^. Making sure that there are representative images from multiple decades of peak fame is required if one wishes to establish a temporal gradient in performance.

As the commonest error type in people with AD is no response^35^, the stage at which PNA occurs cannot easily be determined by an error analysis alone. When there is a failure to name, the standard approach is to ask a series of structured questions to identify at which point naming has broken down. Most researchers adopt a consecutive approach, testing facial recognition, person-based semantics and then naming, although approaches differ. When attempting to calculate the relative weighting for the failure at each stage, it is important to use age-matched control group data. This is because it is not reasonable to expect 100% performance in any face naming task.

Despite its likely ubiquity, PNA remains a relatively underexplored feature of AD, and there have been no reviews of the literature examining the association between AD and PNA and its potential rehabilitation. This is the first systematic review of the topic. Our aims are to:

- Summarise the current literature on PNA in AD with a focus on which components of the cognitive model of naming are responsible when there is a failure to name familiar people.
- Review the claim that PNA is an early behavioural marker of AD.
- Review and evaluate the effectiveness of interventions and support strategies, such as cognitive training, in improving PNA caused by dementia

## Results

### Study selection

**Fig. 2:**
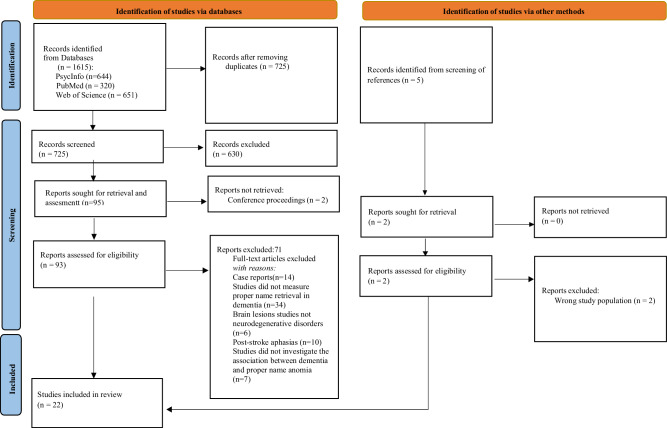
PRISMA flow diagram of the studies in people with PNA and AD.

A total of 1615 studies were retrieved from the electronic databases. After removal of duplicates and applying exclusion criteria based on title and abstract screening, 93 full-text studies were assessed for eligibility. Finally, 22 studies fulfilling all inclusion criteria were included in the review. Summaries of this process are presented in Fig. 2.

### Study characteristics

The characteristics of the 22 studies included in this review are summarized in Table 1 and Table 2. Table 1 deals with the 12 studies that have focused on the nature of PNA in AD, while Table 2 summarizes the 10 interventional studies. All studies employed quantitative methods. Geographically, the studies spanned the following countries. Including the United Kingdom (45%; 10/22), the United States (23%; 5/22), Italy (9%; 2/22), Spain (9%; 2/22), Australia (5%; 1/22), Austria (5%; 1/22), and one study was jointly conducted in Canada and Belgium (5%; 1/22). Participants were predominantly female (60%, as reported in 13/22studies) and had a mean age of 75 (range: 58–87). Dementia severity varied, with mild dementia being the primary focus (50% of studies), followed by moderate (38%) and severe (12%). This distribution highlights the importance of examining PNA at different stages of cognitive decline.

**Table 1:** Summary of eligible studies investigating PNA in people with AD

Authors, Year	Country	Number of participants	Face test	Anomia components tested	Main findings
Hodges et al. (1993)	UK	22 AD patients, 25 healthy controls	Famous Faces Test	Recognition, Naming, Identification, Naming with Semantic Cues, Phonemic Cueing	Sets out the three main stages where proper naming can fail: facial recognition, person-specific semantics and proper name retrieval.
Greene and Hodges (1996)	UK	33 AD patients, 30 healthy controls	Famous Faces Test, Famous Names Test	Recognition, Naming, Identification	Emphasises proper-name deficits at the semantic stage. A temporal gradient was observed where AD patients are better with famous faces from more distant decades.
Greene and Hodges (1998)	UK	24 AD patients, 30 healthy controls	Famous Faces Test	Recognition, Identification, Naming	Semantic information is crucial for being able to retrieve a proper name.
Beeson et al. (1997)	USA	27 AD patients, 33 aphasic stroke patients	Famous Faces Test	Recognition, Identification, Naming, Tip of Tongue state	Moderate AD patients were more impaired than anomic stroke patients in providing semantic information when in a Tip of the Tongue state.
Thompson et al. (2002)	UK	22 AD patients, 31 healthy controls	Famous Faces Test	Recognition, Identification, Naming, Temporal gradient	Deficits in person-specific semantic knowledge and general semantic memory were observed in probable AD patients. Semantic and phonemic cues are less useful for AD patients.
Delazer et al. (2003)	Austria	19 AD patients, 24 MCI patients, 20 healthy controls	Famous Faces Test	Recognition, Identification, Retrieval	AD patients provide significantly less semantic information compared to controls; MCI and Controls perform similarly in name retrieval.
Semenza et al. (2003)	Italy	70 AD patients, 47 healthy controls	Famous Faces Test, Naming Faces, Naming People on Definition	Naming	Evidence that PNA is more sensitive to mild dementia diagnosis than standard long-form tests of dementia.
Estévez-González et al. (2004)	Spain	27 MCI-AD patients, 26 MCI patients, 17 healthy controls	Famous Faces Test	Recognition, Naming,	Severe impairment in recognition of famous faces in the preclinical AD phase; potential as an early marker for AD.
Calabria et al. (2012)	Italy	23 AD patients, 23 healthy controls	Famous Faces Test, Face-name priming task	Naming, semantic categorization	Semantic deficits were observed in PWD through the priming experiment; clarity was lacking in naming tasks due to protocol issues.
Pal et al. (2019)	UK	42 AD patients	Recognition, Naming, Assigning relationship	Recognition, Naming	The estimated facial recognition ability in AD patients is around 33%, potentially inflated due to the lack of a control group.
Garcia et al. (2020)	USA	25 MCI-AD patients	Famous Faces Test	Identification, Naming	MCI patients converting to AD perform worse on the Famous Faces Naming Test compared to non-converters, suggesting utility for early diagnosis.
Gomes et al. (2024)	Spain	35 AD, 50 healthy controls	Famous Faces Test	Recognition, naming, and semantic categorization	AD patients showed impairments in all stages, but difficulties in retrieving proper names mainly reflected degradation at the semantic level; the AD group had more “don’t know” responses, fewer semantic errors, and less benefit from semantic and phonological facilitation than control

**Table 2:** Summary of eligible studies investigating effectiveness of PNA therapy in people with AD

Authors, Year	Country	(N)/ Diagnosis/ Severity	Study design/ Therapy Approach	Materials Used in the Face Learning Task	Duration	Outcome measures	Key findings
Kesslak et al. (1997)	USA	11 AD mild/ moderate 11 controls	Pre-post cohort design with carers acting as controls. Face name rehearsal training	Photographs of group members with associated names and personal background info (novel face-name associations)	4 weeks, group sessions	Face-name recall, Geriatric Depression Scale, digit copying task	Significant improvement in face-name recall and some neuropsychological measures
Clare et al. (1999)	UK	1 AD mild	SCED. Spaced Retrieval, Errorless learning with mnemonic, written and phonological cues	Polaroid photographs of 14 club members	18 sessions over 8 weeks	Relearning, Free name recall	Significant improvement in recalling face-name associations, sustained up to nine months post-intervention in real-life settings
Clare et al. (2002)	UK	12 probable AD mild/ moderate	Pre-post cohort with multiple testing points; item-controlled. Spaced Retrieval, Errorless learning with mnemonic, written and phonological cues	6 photographs of the faces of people known to the participants	6 weekly sessions with self-directed practice in- between sessions	Relearning, Free name recall	Significant improvement in recalling trained faces, maintained some gains at 6- and 12-month post-intervention, and scores above baseline. Only half of the participants responded well to the therapy.
Loewenstein et al. (2004)	USA	44 probable or possible AD, mild	Group randomised (RCT), Cognitive Rehabilitation vs. Mental Stimulation	10 Photographs of staff members (novel face-name associations)	24 sessions of ~45 minutes over 12–16 weeks	Relearning, Free name recall,	Significant improvement in face-name recall and personal information recall in the intervention group
Bier et al. (2008)	Canada/ Belgium	15 AD, mild/15 controls	Pre-post cohort; Spaced Retrieval, Errorless Learning, Vanishing Cues, Trial-and-Error (x2)	5 photographs (novel face-name associations) per participant from a pool of 25	5 weeks, 5 × 45-minute sessions	Free face name recall, cued recall, recognition, error production	All five methods were effective
Hawley et al. (2008)	USA	12 AD mild/ moderate	Group randomised (RCT). Spaced Retrieval vs Uniform Expanded Retrieval	Nine photographs of people (novel face-name association), then a real person	3 weeks, 9 × 1-hour sessions on alternate days	Free recall of pictures and also of real people	Spaced retrieval led to significantly better retention and transfer to live person naming than uniform retrieval
Laffan et al., (2010)	UK	20 possible or probable AD, mild/ moderate	Cohort study, item-randomised: non-learning control, traditional errorless learning, cued errorlesslearning	Famous Faces, names with written and spoken cues	5 weeks, 2 sessions/week, 3 learning conditions	Cued recall task, learning rate, and the effect of cognitive	Self-generated errorless learning led to superior cued recall over traditional errorless learning; patients with higher cognitive function benefited more
Cherry et al. (2010)	UK	4 probable AD, mild/ moderate	Descriptive cohort study Spaced retrieval, Phonological cues	9 colour pictures of male and female adults	9 sessions over 3 weeks	Name retrieval accuracy, retention	Numerical improvement in proper name retrieval accuracy; retention maintained over two weeks
Hopper et al. (2010)	Australia	30 AD, 2 VD, mild/ moderate/ severe	Pre-post cohort, Spaced retrieval, Phonological and semantic cues	4 face-name associations (2 new, 2 previously known)	10 sessions over 2 weeks	Name recall performance, generalization to daily life	Learning efficiency of spaced retrieval therapy; better for previously known associations compared to new ones. Phonological > semantic cueing.
Haslam et al. (2011)	UK	15 dementia (7 AD, 5 VD, 3 mixed) 30 ABI, 60 controls	Cohort study, item-randomised: Trial-and-Error vs. Errorless Learning vs. Spaced Retrieval	6 sets of 10 faces and six lists of 20 common first names	10 sessions	Naming abilities, semantic memory	Spaced retrieval is more effective than errorless learning and trial-and-error for face-name associations in AD and ABI patients.

*ABI* acquired (traumatic) brain injury, *AD* Alzheimer’s Disease, *RCT* randomised clinical trial, *SCED* single-case experimental design, *VD* vascular dementia.

### Narrative syntheses

Across the 22 studies meeting the criteria for inclusion, twelve studies (55%) focused on the association and nature of PNA in AD, with the remaining ten (45%) on treatment. In relation to this first group, most studies investigated deficits in person-specific semantic knowledge and general semantic memory observed in probable AD patients^30,31,36–38^. Other studies highlight the difficulties in face recognition, naming, and semantic memory that are specific to PNA^39,40^. The vast majority of studies included in the review used a version of the Famous Faces Test to measure PNA in people with dementia. This test is sensitive to both cultural context (not many people are famous everywhere) -so its contents vary across geographical regions, and the vicissitudes of time (even fewer are famous forever). Some studies leverage this property of fame by systematically examining the temporal gradients observed in people with dementia, e.g., suggested by Thompson et al.^31^, in 2002.

All studies in this review investigated several key areas related to PNA, with overlapping themes. The result of the most frequently explored topics included: Recognition and retrieval of famous names and faces (55%; 12/22 studies); the impacts of semantic and phonemic cueing (23%; 5/22); associations between name retrieval deficits and dementia progression (45%; 10/22) and the diagnostic utility of PNA in early dementia (23%; 5/22). These studies highlight the importance of proper name retrieval as a marker of disease progression and a potential therapeutic target. (Table 1 and Table 2).

When attempting to calculate the relative weighting for the failure at each stage, it is important to use control group data. This is because as it is not reasonable to expect 100% performance in any face naming task. For example, one study estimated the facial recognition failure rate in people with AD to be 33%, which is likely an overestimate^41^. Six of the reviewed studies included age-matched controls^38,39,42–45^. By subtracting control error rates from the patients’ error rates, we get the following averages (all studies treated equally, regardless of the number of participants). As the data pool is small, we include ranges as a measure of variance. Breakdown of facial recognition was 19% (12–25), person-specific semantics at 30% (10–42), and proper name retrieval 40% (38–48). We will discuss each stage in turn.

Case series of people with posterior cortical atrophy (PCA) are rather small, with prosopagnosia encountered in about 25% of cases^46^, not too dissimilar to the 19% identified in the five studies we found. While classical AD and PCA present with different symptom complexes, in later stages they start to overlap; perhaps this is no surprise given that the majority of PCA cases are due to Alzheimer’s pathology^47^.

Failure at the semantic stage (knowledge of who the person is) is reasonably common, indeed Hodges ^39^ contended this is the primary cause in AD, despite finding equal proportions of failure with semantics or retrieval (40%). They argued this on the basis that semantic cueing didn’t aid proper name retrieval in the majority of subjects; however, semantic cueing is rarely helpful when people are in a Tip-of-the-Tongue (TOT) state, when phonological cueing is generally more effective^48^. Indeed, AD patients in their study clearly benefited from phonemic cueing. In a study contrasting TOT states in people with AD and those with post-stroke aphasia, the stroke group provided significantly more semantic information about unnamed faces than the AD group, confirming that person-specific semantics are more often damaged in people with AD^49^. A study comparing PNA in an AD group with a matched MCI group found a similar pattern^37^. An experiment using face-name priming also found semantic deficits in the AD group, although the results were somewhat confounded due to methodological limitations whereby participants were provided with the famous person’s real name before semantics were probed^50^.

The most recent study of where naming breaks down^45^, used a rather unusual set of famous faces. The age-matched controls only recognised 22% of the sample images as of famous people (from a three-way choice, with two matched foils). Of these, they could only freely name half of them (12% of the stimuli). The AD group recognised a similar percentage (18%), but could only free name 4% of the stimuli. When helping participants name those that they recognised, both groups benefited more from phonological than semantic cues, pointing to word retrieval as the more common locus of failure.

Across the six relevant studies, the commonest point of failure was at the name retrieval stage. People with AD seem to experience a TOT state much like control subjects. Phonemic cueing helps resolve this, but it is less successful than in age-matched controls or those with MCI^37,45,49^, suggesting that there is either some degradation of phonemic knowledge in AD and/or that their retrieval impairments are more severe. The implications for rehabilitation are generally positive as practice-based treatments are considerably more successful for word retrieval than for facial recognition^51,52^ or semantic loss^53^.

#### PNA as an early indicator of AD

As mentioned in the introduction, names for people are harder to retrieve than for other lexical items; indeed, there seems to be an age-related drop-off specifically for proper names^54^. So, it seems reasonable to ask whether PNA might be a good early cognitive indicator for those likely to progress to AD.

Thompson and his research team^31^ studied 31 people with AD, 28 with QAD (‘questionable’ AD, equivalent to MCI, e.g. mean MMSE 27.4/30), and 31 age-matched controls. They wanted to see if faces were more susceptible to the progression of AD than objects. They chose the Graded Naming Test for objects, which is designed to avoid ceiling effects in control subjects^55^. In order to match the face stimuli for difficulty, they created a Graded Faces Test by asking 52 control subjects to freely name 250 pictures of more and less famous people. While the AD group were similarly impaired on naming objects and faces compared with controls, the QAD group were significantly worse than controls at faces than objects. Six out of seven participants who progressed to AD over the next 1–2 years had a relative weakness in face naming, while 17 of the nonconverters scored normally on the Graded Faces Test. This study suggests that PNA may be an early symptom of AD.

Semenza^37^ studied 70 people with confirmed AD, split into three groups based on their MMSE scores; 15 were in the ‘very mild’ group (MMSE range 26–30). For this group, PNA (elicited either by a picture of a famous person or having to name to description) was clearly significantly impaired relative to controls (controls averaged 94% correct, very mild AD averaged 59%)^37^. This is a promising result, but the number of subjects was too small to allow for an accurate calculation of the test’s sensitivity and specificity. Estévez-González^56^ studied two groups of people with an initial diagnosis of MCI. Two years later, 26 retained their MCI status while 27 progressed to AD. There were differences in baseline performance on the famous face naming test, with those who progressed to AD significantly worse than those who didn’t, but the variance across individuals was high, and again, no calculation of specificity or sensitivity was recorded^56^. García et al.^57^ carried out a similar study using the famous faces test, testing 17 people with MCI who remained so and eight who progressed to AD at two years. There was a significant difference between the two groups, but the effect size was small (Cohen’s *d* = 0.42)^57^.

In summary, while PNA may be a good indicator of early AD, no study to our knowledge has examined the sensitivity and specificity of this marker, and in particular, no study has looked at whether it adds any sensitivity and specificity to the currently used, neuropsychometric diagnostic tests.

#### Therapeutic interventions

Across the 22 featured studies, ten (45%) investigated therapeutic approaches for PNA (Table 2). The majority focused on training novel face-name associations rather than previously established ones. Indeed, the earliest study did just this: 11 participants with mild to moderate AD all had to learn each other’s names^58^. The group met weekly for four weeks, with the participants instructed to take the photos and personal information sheets home to review with their carers (who acted as a control group), and to test each other during the intervening days. The carers learnt the names quickly ( ~ 80% correct free recall at one week) while the AD participants’ scores increased in a linear fashion over time, suggesting that, at least over short periods of time, new face-name associations can be learnt by people with AD. Unfortunately, there was no follow-up testing. Loewenstein and colleagues carried out a similar study in 25 people with mild AD, which included learning 10 novel face-name associations as well as training in other cognitive domains, including object recall training, functional tasks, orientation to time and place, and visuo-motor speed of processing. There was a control group (*n* = 19) who undertook practice on computer games of a similar duration^59^. Both groups improved on free recall of names, but there was a significant time*group interaction favouring the rehabilitation group. Importantly, this effect remained significant at the three-month post-therapy testing point.

Two studies using novel face-name stimuli investigated whether it matters which training technique is used. Hawley et al.^60^ compared adjusted spaced retrieval (the spacing of testing was adaptively altered according to whether correct responses were made) vs. uniform expanded retrieval (where the spacing of testing increased uniformly, regardless of naming performance). 12 people with mild to moderate AD were randomly allocated to each group. The stimuli were nine photographs of members of staff, and as well as training and being tested on these, participants were also tested to see if performance transferred to free naming of the live members of staff who came into the room for a ‘surprise’ name recall test. The adjusted spaced retrieval group did significantly better than the uniform expanded retrieval group (67% correct vs. 55% after three weeks of training), but transfer scores to real-world naming of the actual people were very low, with the commonest score being 0/9 in both groups. There was no follow-up time point. Another study looking at novel face-name learning in people with AD systematically varied five different training techniques: spaced retrieval, vanishing cues, errorless learning, and two variants of trial-and-error learning, while they learnt five novel face-name associations^61^. The authors found that all five learning methods were effective, particularly in the immediate post-training phase; however, there were no significant differences in efficacy between the five methods. Delayed recall performance (two weeks post-training) was, not surprisingly, poor with AD participants recalling between 0 and 27% of the trained items. This strongly suggests that training on previously established face-name associations (e.g. prior to AD diagnosis) will be more effective than training on new ones.

Haslam et al.^62^ compared the relative effectiveness of errorless learning vs. spaced retrieval vs. trial-and-error learning in a novel face-name learning paradigm in three separate experiments across three cohorts (young controls, people with acquired (traumatic) brain injury and people with AD). They found that errorless learning and spaced retrieval produced greater accuracy in free recall than trial-and-error learning, but recall under conditions of spaced retrieval was significantly better than that under errorless learning. Cherry et al.^63^ also found positive effects for spaced retrieval in a small study with four people with AD and PNA. Laffan and colleagues^64^ wanted to see if traditional errorless learning techniques could be enhanced by more active engagement of the person with AD, by using cues. They allocated 45 famous face stimuli that each participant recognised but couldn’t name, and randomly allocated these to one of three training sets: non-learning control set, traditional errorless learning set, and a self-generated errorless learning set. The non-learning condition consisted of presenting the pictures of the famous people and asking the participants to guess the names with no feedback given. The traditional errorless learning condition followed a standard procedure, with participants asked to name only if they were certain they knew the correct answer. If they were not sure, they were given the correct name in written form. The self-generated errorless learning condition followed the same procedure, except that the participants were provided with the graphemic cues and asked to guess. If an incorrect response was given at that point, the correct answer was given immediately, orally, by the researcher and the participant was asked to repeat this. There was a significant effect of training type: non-learning < traditional errorless learning < cued errorless learning, with cuing best.

A promising, very detailed single case study in a person with AD and PNA^65^ led Clare et al.^24^ to try out the technique that they had developed with this subject on a cohort of 12 people with PNA caused by AD. In contrast to many of the studies discussed, they personalised the therapy materials to pictures of people in the participant’s social circle and used behaviourally matched famous faces as a within-subject control. The intervention was carried out over six sessions using vanishing cues and spaced retrieval. Unusually, there was a long, post-intervention period of multiple assessments at 1, 3, 6 and 12 months. They found a significant effect on trained items vs. untrained (famous) control items that persisted for at least 6 months. Significant improvement was observed only for the trained familiar faces, with no change for the famous face controls. Two of the 12 participants showed no improvement^24^.

In summary, the evidence suggests that people with PNA caused by AD can learn novel face-name associations and re-learn previously established ones using mass practice-based techniques. Novel associations are, not surprisingly, much harder to learn and seem more vulnerable to temporal degradation. Cued errorless or error-reducing learning paired with spaced retrieval seems to be the most promising approach, with longer-term retention of training effects likely supported by some interval practice. Not surprisingly, most studies found variability in effectiveness across individuals. A proportion of this variability relates to individual characteristics such as baseline cognitive status. Those with better-preserved episodic memory or milder cognitive decline tend to benefit more from mass practice-based interventions, as shown in studies by Bier et al. (2008)^61^ and Laffan et al. (2010)^64^.

#### Gaps and areas for future research

While these studies in the review offer valuable insights, gaps remain. Most studies were geographically concentrated in Western countries, limiting generalizability to diverse populations. Furthermore, the lack of longitudinal data prevents an understanding of the long-term efficacy of therapeutic approaches. Future research should also explore the integration of multiple cueing techniques to maximize intervention outcomes.

Another limitation of the included intervention studies is that studies did not analyze the influence of AD severity on the nature of naming deficits, as the sample was relatively small in our review and could not be divided into subgroups according to the level of cognitive impairment. Therefore, future research should ideally involve RCTs with larger numbers with long-term follow-up or, at the least, longitudinal cohort studies to analyze the ability to retrieve proper names of individuals and how that relates to different stages of dementia. This could provide a better understanding of the nature of the deficit in retrieving proper names across the course of dementia and what interventions may be appropriate at each stage.

## Discussion

This is the first systematic review focusing on PNA in AD. We found 22 research articles, ten of which were interventional; the remainder were observational. We attempted to answer three questions: a) within a standard neuro-cognitive model, impairment of which cognitive process might cause PNA (facial recognition, person-specific semantics or proper name retrieval)? b) Is PNA a useful diagnostic sign of emerging AD? and, c) Can practice-based rehabilitation improve symptoms of PNA in AD? We summarise our main findings below and conclude the discussion with a section on clinical ramifications.

Regarding the cognitive locus of failure to name a familiar person, we found studies that highlighted the three main processes involved, namely: facial recognition, “I recognise that face”; semantic knowledge, “I know who they are”; and proper name retrieval, “I can say their name.” Unless there are other clinical features that suggest a breakdown in visual processing, facial recognition is the least common cause across the studies (19%). Indeed, if present, we suggest systematic visual testing to see if the phenotype is more in keeping with posterior cortical atrophy rather than classical AD. Semantic knowledge can become eroded in AD, and this was the next most common locus (30%). Some care needs to be taken with the materials used because of the temporal gradients seen in AD. It is worth checking that stimuli are appropriate to the person in question. This means selecting images of a range of people whose fame peaked over several different decades. One such study showed that controls were significantly better at naming people from the 1980s compared with those from the 1940s, while people with AD showed the reverse pattern^38^. The authors concluded that this was most likely due to AD resulting in an impairment of episodic memory (so not encoding more recently famous people) rather than a temporally mediated degradation of semantic memory. Other studies have replicated this finding, but enough time bins must be used. Delazer et al.^37^ found a temporal gradient with three-time bins, whereas Thompson only employed two ^31^. Lastly, proper name retrieval was the commonest cause of PNA (40%); this is likely the case because most people with symptomatic AD have an aphasic disorder, even early on in the disease process and especially if language is examined beyond the single word level^66^. Allied to this, most people find proper names harder to produce than any other noun category^19^, so it is understandable that this becomes amplified in people with AD and word-finding difficulties.

There are hints that in some people, PNA may indeed be a harbinger of incipient AD, but to date, there have not been large enough studies to allow for a proper evaluation of the sensitivity and specificity of this, or whether it adds predictive power to existing behavioural or biological markers. As diagnostic frameworks increasingly incorporate biomarkers, future studies should assess whether PNA adds any additional sensitivity in diagnosing Alzheimer’s disease. The utility of PNA as a diagnostic tool for early AD, therefore, requires further investigation.

We identified ten studies that investigated whether PNA is a realistic therapeutic target for behavioural intervention. The most successful studies used techniques such as errorless learning, mnemonic cueing and spaced retrieval. These require minimal cognitive effort, are non-invasive, and can be implemented by caregivers or support staff across a range of settings, including home care and residential facilities. The integration of active participation elements, such as self-generation, further enhances their utility without necessitating complex tools or professional expertise. Some people with AD can learn novel face-name associations, but it takes a lot of repetitions, leaves them prone to errors and does not seem to have long-lasting effects. By contrast, relearning or re-establishing face-name association for people that they previously knew is more effective in about 50% of cases, with practice-based effects persisting in some cases for up to a year. As the cause of PNA is often at the retrieval stage, cueing is effective. Semantic and phonological cues seem to work, with phonological cueing being a bit more effective on average. Employing an errorless learning approach (e.g. vanishing cues) and combining this with spaced retrieval seems to work best.

Pinpointing the main cognitive locus of PNA helps with differential diagnosis and has ramifications for rehabilitation. In a busy service such as a memory clinic, however, it can be difficult to make time for individualised testing. While standardised tests are the default in neuropsychological assessment, this is not possible with any Famous Faces test as fame varies across time and has a strong cultural bias. Therefore, when testing any patients who might have PNA, we recommend keeping some pictures of relevant famous people handy (both currently and previously famous), with an eye on cultural relevance to the patient. The easy availability of images on internet search engines makes this a much more tractable task. Failure at the recognition level is difficult to take forward without specialised testing, but those with acquired prosopagnosia are usually aware that they are better at recognising people from their voices, so they enquire about this. There are some very sensitive tests for prosopagnosia, such as the Cambridge Face Memory Test, which involves learning digitally manipulated images of faces and then recognising them from novel views and with added visual noise, but they require around 45 min to complete^67^.

Those with semantic problems will fail to give detailed information about the people whom they recognise from their image. Whether encountered in AD or semantic dementia, semantic symptoms will affect other domains, so it is worth testing this with a short, semantically-based test such as the Pyramids and Palm Trees Test^68^, or the quicker to administer Repeat and Point test^69^. Lastly, if name retrieval is the root cause, then prompting with either phonological or semantic cues should help establish this.

Cognitive rehabilitation is emerging as a valuable tool to help people with dementia relearn the names of people they have struggled to remember. Studies provide initial indications (through case studies and series) that those in the early stages of AD may potentially benefit from such interventions. Further longitudinal RCT evidence is needed. Recommended strategies for families include using memory books or photo albums with names, keeping a personalised name book with photographs, and regularly showing it and practising the names to reinforce recall and face-name associations. We have just finished conducting just such a trial with a digital therapeutic app called Gotcha! Subjects enter pictures of those they wish to get better at being able to name. The therapy algorithm uses vanishing phonological cues and spaced retrieval principles and has a novel utterance verifier to drive progression through the therapy pathway^70^. The trial will report in 2026: https://www.ucl.ac.uk/brain-sciences/icn/research/research-groups/neurotherapeutics/leff-lab/leff-lab-therapy-apps/gotcha.

## Methods

The systematic review was carried out following the PRISMA reporting guidelines for systematic reviews throughout the current paper. Searches were conducted between August 2024 and February 2025.

### Eligibility criteria

Included studies had to focus on adults (18+), report quantitative measures of PNA (e.g., Famous Faces Test, Famous Names Test) and have separate reporting on a group of people with AD (diagnosed based on clinical diagnosis), and look either at prediction of Alzheimer by PNA or differences in PNA between AD and control, or rehabilitation of PNA in AD. All included studies relied on clinical diagnostic criteria; none employed biomarker confirmation, such as amyloid PET or CSF analysis.

Non-peer-reviewed papers, case reports, editorials, commentaries and uncontrolled studies were excluded due to their limited generalisability. Finally, only peer-reviewed, original research papers and only studies written in English were included in our final assessment.

### Information sources, search strategy and databases

Searches were conducted in PubMed, PsycInfo and Web of Science databases. Any systematic reviews that were identified were also re-screened for relevant references. Filters added to PubMed searches were restricted to human studies only.

Search terms for AD and PNA were constructed using a combination of keywords and spelling variants. Search terms adapted to the database were reviewed by the senior researcher to confirm that the terms met the study’s objectives and accurately captured relevant literature (Supplementary Note 1: Detailed database search strings).

Search terms included (PNA OR anomia OR “people’s names” OR “proper names”)** AND*(Alzheimer OR “Alzheimer’s disease” OR “people with dementia”)**. To identify studies related to interventions, the following treatment-related terms were included: (treatment OR therapy OR rehabilitation* OR “errorless learning” OR “spaced retrieval” OR “cognitive training”) **. The search was limited to articles written in English and studies involving human participants. Once an initial search of titles and abstracts was completed, the reference list of articles was also searched (see Fig. 2)

### Selection process

Records were first screened for duplicates using EndNote, which was followed by manual removal (Clarivate Analytics). The inclusion and exclusion criteria were used at each stage (title, abstract and full-text review, reference search). The full text of studies identified from the databases was independently screened by two reviewers (AB & AL) against the eligibility criteria. Any disagreements were resolved by the third reviewer (JS) by discussion, and the Newcastle–Ottawa Scale was used to assess risk of bias and evaluate the methodological quality of the included studies. Potential studies with relevant content were completely read and evaluated for inclusion, and further screened the references of these articles for missing relevant publications.

### Data collection process and data items

The following data from eligible studies were then extracted by the two reviewers independently, and data extraction was completed using a custom-made form. All extracted data was then cross-reviewed, and any missed information was added. Study characteristics: Study authors, year of publication, country study was conducted, study design, recruitment method, allocation method (if applicable). Participant characteristics: Inclusion/exclusion criteria. Task characteristics: Description of PNA, diagnosing method and naming task used. Outcome information: Main results (if applicable), intervention results (if applicable). Any disagreements about inclusion were discussed between AB and AL.

Full-text screening was then performed on all potentially relevant records that could be retrieved by the same two reviewers, also independently. All retrieved articles were read in full and assessed for eligibility using a pre-determined inclusion checklist. Any inconsistencies between reviewers (at any stage of the screening process) were resolved through discussion, and any disagreements were resolved by discussion.

### Treatment of PNA

All relevant interventions described in the studies were reviewed. However, a statistical meta-analysis was not conducted due to the high heterogeneity of the study methodologies. Instead, a narrative synthesis of the results was performed as per Popay et al.^71^ with the review aims used to derive the thematic structure of the results.

We describe the main characteristics of the 22 key papers (Supplementary Table 1) in the results section and discuss their contents and key themes in the discussion section. We finish with a section on clinical diagnosis and management of PNA.

### Ethical approval statement

This study is a systematic review of previously published literature and does not involve human participants or primary data collection. Therefore, ethical approval is not required.

## Supplementary information

Supplementary Information

## Data Availability

No new datasets were generated or analysed during the current study.
