# Supplementary material for: Proper name anomia in people with Alzheimer’s disease: implications for diagnosis and treatment—a systematic review
Source: NPJ Dement. 2026 Feb 2;2(1):11. doi: 10.1038/s44400-026-00058-y (PMC12864043; doi:10.1038/s44400-026-00058-y)
Supplement: Supplementary file 1 — Supplementary Information [file 44400_2026_58_MOESM1_ESM.pdf]

Supplementary Note 1. Detailed database search strings

1. PubMed

- Search Terms:
  - "PNA" OR "anomia*" OR "people's names*" OR "proper names"
  - "dementia" OR "Alzheimer's disease*" OR "people with dementia"

2. PsycInfo

- Search Terms:
  - "PNA" OR "anomia*" OR "people's names*" OR "proper names"
  - "dementia" OR "Alzheimer's disease*" OR "people with dementia"

3. Web of Science

- Search Terms:
  - "PNA" OR "anomia*" OR "people's names*" OR "proper names"

"dementia" OR "Alzheimer's disease*" OR "people with dementia"

PRISMA

PRISMA 2020 Checklist

Section and Topic	Item #	Checklist item	Location where item is reported
**TITLE**			
Title	1	Identify the report as a systematic review.	Title page ("PNA in People with Alzheimer's Disease: Implications for Diagnosis and Treatment – A Systematic Review")p.1
**ABSTRACT**			
Abstract	2	See the PRISMA 2020 for Abstracts checklist.	Abstract, p. 2 (single paragraph, ≤150 words)
**INTRODUCTION**			
Rationale	3	Describe the rationale for the review in the context of existing knowledge.	Introduction, p 3
Objectives	4	Provide an explicit statement of the objective(s) or question(s) the review addresses.	Introduction p 6-7
**METHODS**			
Eligibility criteria	5	Specify the inclusion and exclusion criteria for the review and how studies were grouped for the syntheses.	P31
Information sources	6	Specify all databases, registers, websites, organisations, reference lists and other sources searched or consulted to identify studies. Specify the date when each source was last searched or consulted.	P33
Search strategy	7	Present the full search strategies for all databases, registers and websites, including any filters and limits used.	P33
Selection process	8	Specify the methods used to decide whether a study met the inclusion criteria of the review, including how many reviewers screened each record and each report retrieved, whether they worked independently, and if applicable, details of automation tools used in the process.	P34
Data collection process	9	Specify the methods used to collect data from reports, including how many reviewers collected data from each report, whether they worked independently, any processes for obtaining or confirming data from study investigators, and if applicable, details of automation tools used in the process.	P34
Data items	10a	List and define all outcomes for which data were sought. Specify whether all results that were compatible with each outcome domain in each study were sought (e.g. for all measures, time points, analyses), and if not, the methods used to decide which results to collect.	**Outcomes** Naming accuracy, error types, and response latency. Pre- and post-therapy outcomes were extracted.
	10b	List and define all other variables for which data were sought (e.g. participant and intervention characteristics, funding sources). Describe any assumptions made about any missing or unclear information.	**Other variables** Study details (author, year, design, country), participant characteristics (n, age, sex, dementia severity), intervention features (type, duration, frequency), setting, and funding sources. Missing data recorded as "not reported
Study risk of bias assessment	11	Specify the methods used to assess risk of bias in the included studies, including details of the tool(s) used, how many reviewers assessed each study and whether they worked independently, and if applicable, details of automation tools used in the process.	We did not perform a formal risk of bias assessment (e.g., RoB2 or NOS) because of the small number of eligible studies
Effect measures	12	Specify for each outcome the effect measure(s) (e.g. risk ratio, mean difference) used in the synthesis or presentation of results.	As no meta-analysis was undertaken, outcomes were narratively described.No pooled effect estimates were calculated
Synthesis methods	13a	Describe the processes used to decide which studies were eligible for each synthesis (e.g. tabulating the study intervention characteristics and comparing against the planned groups for each synthesis (item #5)).	Studies were grouped by therapy approach (spaced retrieval, errorless learning) and population characteristics

PRISMA 2020 Checklist

Section and Topic	Item #	Checklist item	Location where item is reported
	13b	Describe any methods required to prepare the data for presentation or synthesis, such as handling of missing summary statistics, or data conversions.	Data were extracted into summary tables; no statistical conversions were applied.
	13c	Describe any methods used to tabulate or visually display results of individual studies and syntheses.	Results are presented in structured tables (study design, sample, intervention, outcome)
	13d	Describe any methods used to synthesize results and provide a rationale for the choice(s). If meta-analysis was performed, describe the model(s), method(s) to identify the presence and extent of statistical heterogeneity, and software package(s) used.	Narrative synthesis was performed because of heterogeneity in design and outcomes; meta-analysis was not feasible
	13e	Describe any methods used to explore possible causes of heterogeneity among study results (e.g. subgroup analysis, meta-regression).	Sources of heterogeneity were explored narratively (e.g., intervention length, dementia severity).
	13f	Describe any sensitivity analyses conducted to assess robustness of the synthesized results.	No sensitivity analyses were performed due to the limited number of studies
Reporting bias assessment	14	Describe any methods used to assess risk of bias due to missing results in a synthesis (arising from reporting biases).	No formal assessment of reporting bias was undertaken.
Certainty assessment	15	Describe any methods used to assess certainty (or confidence) in the body of evidence for an outcome.	We did not apply GRADE or a similar framework given the small number of studies and heterogeneity.
**RESULTS**			
Study selection	16a	Describe the results of the search and selection process, from the number of records identified in the search to the number of studies included in the review, ideally using a flow diagram.	P 7
	16b	Cite studies that might appear to meet the inclusion criteria, but which were excluded, and explain why they were excluded.	P7
Study characteristics	17	Cite each included study and present its characteristics.	P 18,19,20,21 ,22,23
Risk of bias in studies	18	Present assessments of risk of bias for each included study.	See above; no structured tool applied, but limitations of each study are described narratively in the Results.
Results of individual studies	19	For all outcomes, present, for each study: (a) summary statistics for each group (where appropriate) and (b) an effect estimate and its precision (e.g. confidence/credible interval), ideally using structured tables or plots.	P 12,13,14,15
Results of syntheses	20a	For each synthesis, briefly summarise the characteristics and risk of bias among contributing studies.	
	20b	Present results of all statistical syntheses conducted. If meta-analysis was done, present for each the summary estimate and its precision (e.g. confidence/credible interval) and measures of statistical heterogeneity. If comparing groups, describe the direction of the effect.	P 14,15,16
	20c	Present results of all investigations of possible causes of heterogeneity among study results.	No formal investigation of heterogeneity was conducted due to the small number and heterogeneity of included studies;
	20d	Present results of all sensitivity analyses conducted to assess the robustness of the synthesized results.	No sensitivity analyses were performed as no statistical synthesis/meta-analysis was undertaken
Reporting biases	21	Present assessments of risk of bias due to missing results (arising from reporting biases) for each synthesis assessed.	No formal assessment of reporting bias was undertaken. .
Certainty of evidence	22	Present assessments of certainty (or confidence) in the body of evidence for each outcome assessed.	We did not apply GRADE or a similar framework given the small number of studies and heterogeneity.

PRISMA 2020 Checklist

Section and Topic	Item #	Checklist item	Location where item is reported
**DISCUSSION**			
Discussion	23a	Provide a general interpretation of the results in the context of other evidence.	P 28,29
	23b	Discuss any limitations of the evidence included in the review.	P29
	23c	Discuss any limitations of the review processes used.	P30
	23d	Discuss implications of the results for practice, policy, and future research.	P31
**OTHER INFORMATION**			
Registration and protocol	24a	Provide registration information for the review, including register name and registration number, or state that the review was not registered.	CRD42023415044 Prospero
	24b	Indicate where the review protocol can be accessed, or state that a protocol was not prepared.	No separate protocol was made
	24c	Describe and explain any amendments to information provided at registration or in the protocol.	No new amendments were made
Support	25	Describe sources of financial or non-financial support for the review, and the role of the funders or sponsors in the review.	NIHR
Competing interests	26	Declare any competing interests of review authors.	No conflict of interest
Availability of data, code and other materials	27	Report which of the following are publicly available and where they can be found: template data collection forms; data extracted from included studies; data used for all analyses; analytic code; any other materials used in the review.	The study-level extracted data from the 22 included studies are provided in Tables 1 and 2 of the manuscript. Additional details are available from the corresponding author upon request.

From: Page MJ, McKenzie JE, Bossuyt PM, Boutron I, Hoffmann TC, Mulrow CD, et al. The PRISMA 2020 statement: an updated guideline for reporting systematic reviews. BMJ 2021;372:n71. doi: 10.1136/bmj.n71.
This work is licensed under CC BY 4.0. To view a copy of this license, visit https://creativecommons.org/licenses/by/4.0/

Supplementary Table 1

Eligible studies investigating the PNA in people with AD

Authors, Year	Country	Number of participants	Face test	Anomia components tested	Main findings
Hodges et al. (1993)	UK	22 AD patients, 25 healthy controls	Famous Faces Test	Recognition, Naming, Identification, Naming with Semantic Cues, Phonemic Cueing	Sets out the three main stages where proper naming can fail: facial recognition, person-specific semantics and proper name retrieval.
Greene and Hodges (1996)	UK	33 AD patients, 30 healthy controls	Famous Faces Test, Famous Names Test	Recognition, Naming, Identification	Emphasises proper-name deficits at the semantic stage. Temporal gradient observed where AD patients are better with famous faces from more distant decades.
Greene and Hodges (1998)	UK	24 AD patients, 30 healthy controls	Famous Faces Test	Recognition, Identification, Naming	Semantic information crucial for being able to retrieval a proper name.

Beeson et al. (1997)	USA	27 AD patients, 33 aphasic stroke patients	Famous Faces Test	Recognition, Identification, Naming, Tip of Tongue state	Moderate AD patients more impaired than anomic stroke patients in providing semantic information when in a Tip of the Tongue state.
Thompson et al. (2002)	UK	22 AD patients, 31 healthy controls	Famous Faces Test	Recognition, Identification, Naming, Temporal gradient	Deficits in person-specific semantic knowledge and general semantic memory observed in probable AD patients. Semantic and phonemic cues less useful for AD patients.
Delazer et al. (2003)	Austria	19 AD patients, 24 MCI patients, 20 healthy controls	Famous Faces Test	Recognition, Identification, Retrieval	AD patients provide significantly less semantic information compared to controls; MCI and

					controls perform similarly in name retrieval.
Semenza et al. (2003)	Italy	70 AD patients, 47 healthy controls	Famous Faces Test, Naming Faces, Naming People on Definition	Naming	Evidence that PNA is more sensitive to mild dementia diagnosis than standard 'long form' tests of dementia.
Estévez-González et al. (2004)	Spain	27 MCI-AD patients, 26 MCI patients, 17 healthy controls	Famous Faces Test	Recognition, Naming,	Severe impairment in recognition of famous faces in preclinical AD phase; potential as an early marker for AD.
Calabria et al. (2012)	Italy	23 AD patients, 23 healthy controls	Famous Faces Test, Face-name priming task	Naming, semantic categorization	Semantic deficits observed in PWD through priming experiment; clarity lacking in naming tasks due to protocol issues.

Pal et al. (2019)	UK	42 AD patients	Recognition, Naming, Assigning relationship	Recognition, Naming	Estimated facial recognition ability in AD patients around 33%, potentially inflated due to lack of control group.
Garcia et al. (2020)	USA	25 MCI-AD patients	Famous Faces Test	Identification, Naming	MCI patients converting to AD perform worse on Famous Faces Naming Test compared to non-converters, suggesting utility for early diagnosis.

Gomes et al. (2024)	Spain	35 AD ,50 healthy controls	Famous Faces Test,	Recognition, naming, semantic categorization	AD patients showed impairments in all stages, but difficulties in retrieving proper names mainly reflected degradation at the semantic level; AD group had more "don't know" responses, fewer semantic errors, and less benefit from semantic and phonological facilitation than control

Authors, Year	Country	(N)/ Diagnosis/ Severity	Study design/ Therapy Approach	Materials Used in the Face Learning Task	Duration	Outcome measures	Key findings
Kesslak et al., (1997)	USA	11 AD mild/ moderate<br><br>11 Controls	Pre-post cohort design with carers acting as controls.<br>Face name rehearsal training	Photographs of group members with associated names and personal background info (novel face-name associations)	4 weeks, group sessions	Face-name recall, Geriatric Depression Scale, digit copying task	Significant improvement in face-name recall and some neuropsychological measures
Clare et al. (1999)	UK	1 AD mild	SCED.<br>Spaced Retrieval, Errorless learning with mnemonic, written and phonological cues	Polaroid photographs of 14 club members	18 sessions over 8 weeks	Relearning, Free name recall	Significant improvement in recalling face-name associations, sustained up to nine months post-intervention in real-life settings

Clare et al. (2002)	UK	12 probable AD mild/ moderate	Pre-post cohort with multiple testing points; item-controlled. Spaced Retrieval, Errorless learning with mnemonic, written and phonological cues	6 photographs of faces of people known to the participants	6 weekly sessions with self-directed practice in-between sessions	Relearning, Free name recall	Significant improvement in recalling trained faces, maintained some gains at 6- and 12-months post-intervention, scores above baseline. Only half of the participants responded well to the therapy.
Loewenstein et al., (2004)	USA	44 probable or possible AD, mild	Group randomised (RCT), Cognitive Rehabilitation vs. Mental Stimulation	10 Photographs of staff members (novel face-name associations)	24 sessions of ~45mins over 12-16 weeks	Relearning, Free name recall,	Significant improvement in face-name recall and personal information recall in the intervention group

Bier et al., (2008)	Canada/ Belgium	15 AD, mild<br><br>15 controls	Pre-post cohort; Spaced Retrieval, Errorless Learning, Vanishing Cues, Trial-and-Error (x2)	5 photographs (novel face-name associations) Per participant from a pool of 25	5 weeks, 5 × 45-minute sessions	Free face name recall, cued recall, recognition, error production	All five methods were effective
Hawley et al., (2008)	USA	12 AD mild/ moderate	Group randomised (RCT). Spaced Retrieval vs Uniform Expanded Retrieval	Nine photographs of people (novel face-name association), then real person	3 weeks, 9 × 1-hour sessions on alternate days	Free recall to pictures and also to real person	Spaced retrieval led to significantly better retention and transfer to live person naming than uniform retrieval

Laffan et al., (2010)	UK	20 possible or probable AD, mild/ moderate	Cohort study, item-randomised: non-learning control, traditional errorless learning, cued errorless learning	Famous Faces, names with written and spoken cues	5 weeks, 2 sessions/week, 3 learning condition	Cued recall task, learning rate, effect of cognitive	Self-generated errorless learning led to superior cued recall over traditional errorless learning; patients with higher cognitive function benefited more
Cherry et al. (2010)	UK	4 probable AD, mild/ moderate	Descriptive cohort study<br>Spaced retrieval, Phonological cues	9 colour pictures of male and female adults	9 sessions over 3 weeks	Name retrieval accuracy, retention	Numerical improvement in proper name retrieval accuracy; retention maintained over two weeks
Hopper et al. (2010)	Australia	30 AD, 2 VD,	Pre-post cohort, Spaced retrieval	4 face-name associations (2 new, 2	10 sessions over 2 weeks	Name recall performance,	Learning efficiency of spaced retrieval therapy;

		mild/ moderate/ severe	Phonological and semantic cues	previously known)		generalization to daily life	better for previously known associations compared to new ones. Phonological > semantic cueing.
Haslam et al. (2011)	UK	15 dementia (7 AD, 5 VD, 3 mixed) 30 ABI, 60 controls	Cohort study, item-randomised: Trial-and-Error vs. Errorless Learning vs. Spaced Retrieval	6 sets of 10 faces and six lists of 20 common first names	10 sessions	Naming abilities, semantic memory	Spaced retrieval more effective than errorless learning and trial-and-error for face-name associations in AD and ABI patients.

Table S1. Newcastle–Ottawa Ratings for Diagnostic / Observational Studies

Reference	Selection (max 5 stars)	Comparability (max 2 stars)	Outcome / Exposure (max 3 stars)	Total (max 10 stars)
Hodges et al. (1993)	4	2	3	9
Greene & Hodges (1996)	4	2	3	9
Greene & Hodges (1998)	4	2	3	9
Beeson et al. (1997)	3	1	3	7
Thompson et al. (2002)	4	2	3	9
Delazer et al. (2003)	3	2	3	8
Estévez-González et al. (2004)	4	2	3	9
Calabria et al. (2012)	3	2	2	7
Pal et al. (2019)	3	1	3	7
Garcia et al. (2020)	3	2	3	8
Gomes et al. (2024)	4	2	3	9
Semenza et al. (2003)	4	2	3	9

Table S2. Newcastle–Ottawa Ratings for Intervention / Therapy Studies

Reference	Selection (max 5 stars)	Comparability (max 2 stars)	Outcome / Exposure (max 3 stars)	Total (max 10 stars)
Kesslak et al. (1997)	4	1	2	7
Clare et al. (1999)	3	1	2	6
Clare et al. (2002)	4	1	3	8
Loewenstein et al. (2004)	5	1	3	10
Bier et al. (2008)	4	1	2	7
Hawley et al. (2008)	5	1	3	9
Laffan et al. (2010)	4	1	3	8
Cherry et al. (2010)	3	1	2	6
Hopper et al. (2010)	4	1	3	8
Haslam et al. (2011)	4	1	3	8
